# Impact of the Current on Reverse Bias Degradation of Perovskite Solar Cells

**DOI:** 10.1021/acsaem.3c02273

**Published:** 2023-11-10

**Authors:** Jonathan Henzel, Klaas Bakker, Mehrdad Najafi, Valerio Zardetto, Sjoerd Veenstra, Olindo Isabella, Luana Mazzarella, Arthur Weeber, Mirjam Theelen

**Affiliations:** † TNO, partner in Solliance, High Tech Campus 21, 5656 AE Eindhoven, The Netherlands; ‡ Photovoltaic Materials and Devices, Delft University of Technology, Mekelweg 5, 2628 CD Delft, The Netherlands

**Keywords:** perovskite solar cells, reverse bias, partial shading, degradation, stability, negative voltages

## Abstract

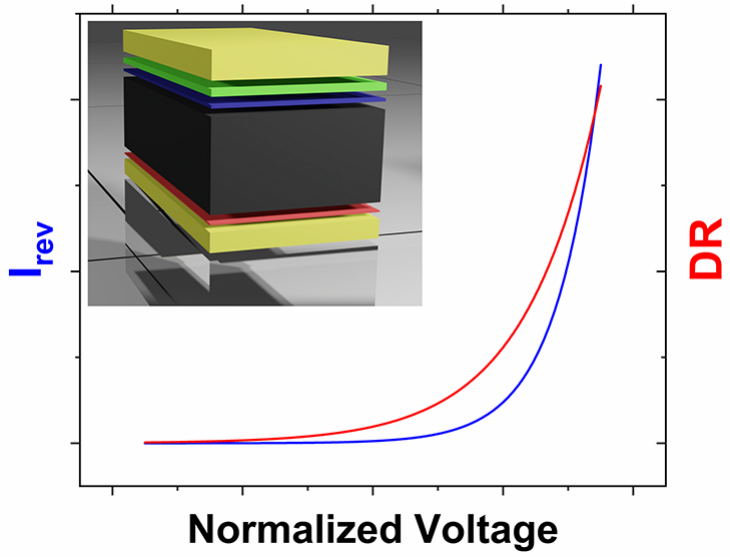

Nonequal current generation in the cells of a photovoltaic module, e.g., due to partial shading, leads to operation in reverse bias. This quickly causes a significant efficiency loss in perovskite solar cells. We report a more quantitative investigation of the reverse bias degradation. Various small reverse biases (negative voltages) were applied for different durations. After normalizing the applied voltages with the breakdown voltages, we found similar dependences of the reverse bias current and the degradation rate. We draw conclusions regarding possible degradation mechanisms and propose a way to increase the comparability of degradation rates for comparing different perovskite solar cells.

The topic of partial shading and reverse bias stability has only recently begun to attract specific interest in the perovskite community.^1^ Progress toward commercialization of the technology is one main driver behind the increased effort dedicated to the issue of stability in general of which research into the reverse bias behavior is a part. Reverse biases hold a special status as stressors that cannot be mitigated by packaging^1^ but need to be researched on the cell level. Despite its relevance, also for silicon–perovskite tandem modules,^2−4^ the field of reverse bias degradation does not receive much attention and detailed mechanisms are still unclear or require validation.

Reverse biases occur due to nonequal current generation in the series-connected cells of a photovoltaic module. Reasons for the occurrence of reverse biases can be partial shading, local differences in aging, and manufacturing defects.^1^ The observed loss in power conversion efficiency (PCE) following instances of reverse bias has been ascribed to various degradation mechanisms. Important phenomenological studies have been published by Bowring et al. and Razera et al., whose observations are the basis upon which most discussed degradation mechanisms are built.^5,6^ An overview over the present state of research is given in Wang et al.^7^ A notable achievement of increased stability was reported by Bogachuk et al. on mesoscopic, carbon-electrode, single-cation, single-halide perovskite solar cells and mini-modules.^8^

In several publications, the role of the current in reverse bias degradation mechanisms has been mentioned. Bowring et al. suggested an electrochemical reaction at an interface to explain a decrease of the reverse bias current over time under a constant reverse bias.^5^ Razera et al. investigated the effects of a voltage below and above the breakdown voltage on halide phase segregation.^6^ Bertoluzzi et al. presented a new degradation mechanism that is directly dependent on the reverse bias current flowing through the cell.^9^ Finally, Ni et al. added a hole blocking layer and reported slower degradation, possibly due to reduced current injection.^10^ On the other hand, the migration of iodide into the organic electron transport layer—as proposed by Razera et al. and further investigated by Gould et al.—can be considered electric-field-driven.^6,11^ The same could be true for the formation of local shunts due to migration of metal ions or destabilizing accumulation of ions.^6,12^

Most of the mentioned publications describe reverse bias degradation only phenomenologically. We contribute a more quantitative investigation that considers the integrated impact of all occurring degradation mechanisms. We found that we can compensate for cell-to-cell differences by normalizing the applied voltage to the breakdown voltage. In this way, we reveal exponential relationships of the reverse bias current and of the degradation rate with the normalized voltage. We discuss how this normalization method can be used to investigate the dominant degradation mechanisms and to increase the comparability of the reported degradation rates.

Our samples were planar perovskite solar cells in the p-i-n configuration with the following layer stack: glass/ITO/PTAA/perovskite/C_60_/SnO_2_/ITO. The absorber was the triple-cation, double-halide perovskite Cs_0.05_MA_0.15_FA_0.8_PbI_2.7_Br_0.3_ with a bandgap of 1.6 eV. The power conversion efficiencies (PCEs) of the best 20 cells used in the following experiments were determined as 15.6 ± 0.1% by maximum-power-point-tracking (MPPT). Further details of the fabrication process and the samples can be found in the Supporting Information on page S1 and Table S1. Parts of the process were based on Bracesco et al.^13^ and Glowienka et al.^14^

Our experimental procedure to investigate reverse bias degradation can be separated into an initial characterization, a degradation step, and a final characterization. The characterization steps consisted of illuminated current–voltage measurements (IV), dark IV measurements (DIV), dark IV measurements with an extended voltage range to investigate the reverse bias response (DIV_ext_), and MPPT. During the degradation step, a constant negative voltage (*V*_appl_) was applied to a cell for a duration *t*_deg_ while the reverse bias current (*I*_rev_) was monitored. With the cell area, we calculated a reverse bias current density *J*_rev_. Among the samples, *V*_appl_ was varied between −1 V and −4 V and *t*_deg_ between 15 min. and 60 min.; in one case, it was extended to 990 min. Further details can be found in the Supporting Information on page S2 and in Table S2.

We use the breakdown voltage *V*_bd_, determined from DIV_ext_ measurements, as a metric to describe the reverse bias behavior of a cell. Following the definition by Bowring et al., we defined *V*_bd_ as the voltage at which an overall current density of −1 mA/cm^2^ flows through the device.^5^ As we observe the hysteretic behavior of the current density in the negative voltage regime, the mean of the breakdown voltages from the forward and reverse sweeps is used. As an example, the result of a DIV_ext_ measurement of a single cell and the determination of *V*_bd_ is depicted in Figure 1. The same is shown for all cells used in these experiments in the Supporting Information on page S4 in Figure S1. The 20 cells showed breakdown voltages between −3.3 V and −5.1 V.

**Figure 1:**
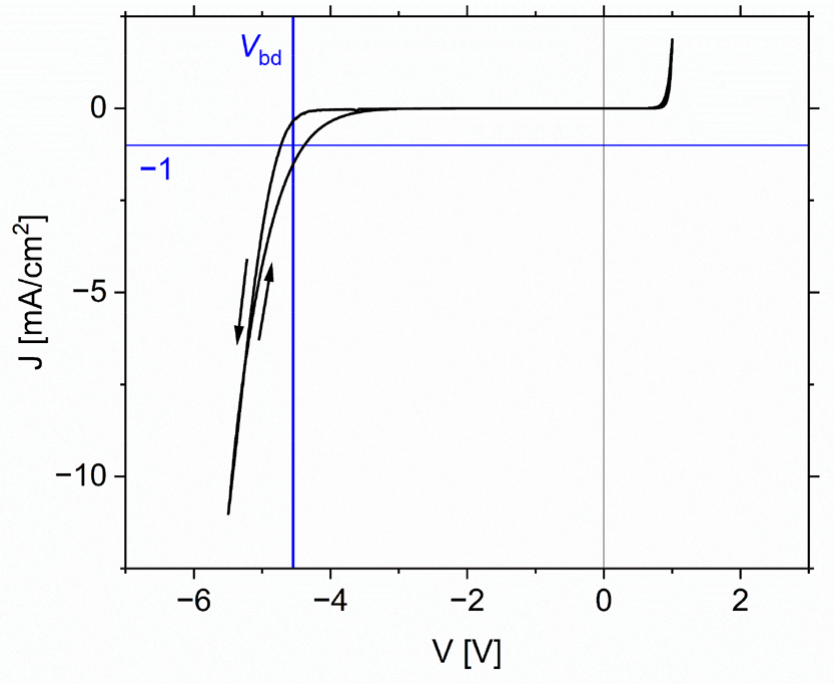
Dark current density–voltage curve in the reverse bias regime of one perovskite solar cell. The horizontal blue line marks the current density of −1 mA/cm^2^, and the vertical blue line marks the breakdown voltage *V*_bd_.

The average *J*_rev,avg_ of the current density *J*_rev_ that flows through a cell during reverse bias degradation is used as a metric to compare the current flow (see Supporting Information on page S3). In the following, we will always consider the absolute of *J*_rev,avg_. It is shown in Figure 2a where we plot *J*_rev,avg_ against the applied voltage. We observe an increase of *J*_rev,avg_ with *V*_appl_ as can be expected from the DIV_ext_ measurements. We, however, also observe a significant spread at large and small reverse biases. We can explain the spread at small negative voltages with the existence of local shunts that form the dominating reverse bias current pathway far below the reverse bias breakdown. For the spread at larger negative voltages, the spread of the breakdown voltage is directly responsible, as will be shown in the following.

**Figure 2:**
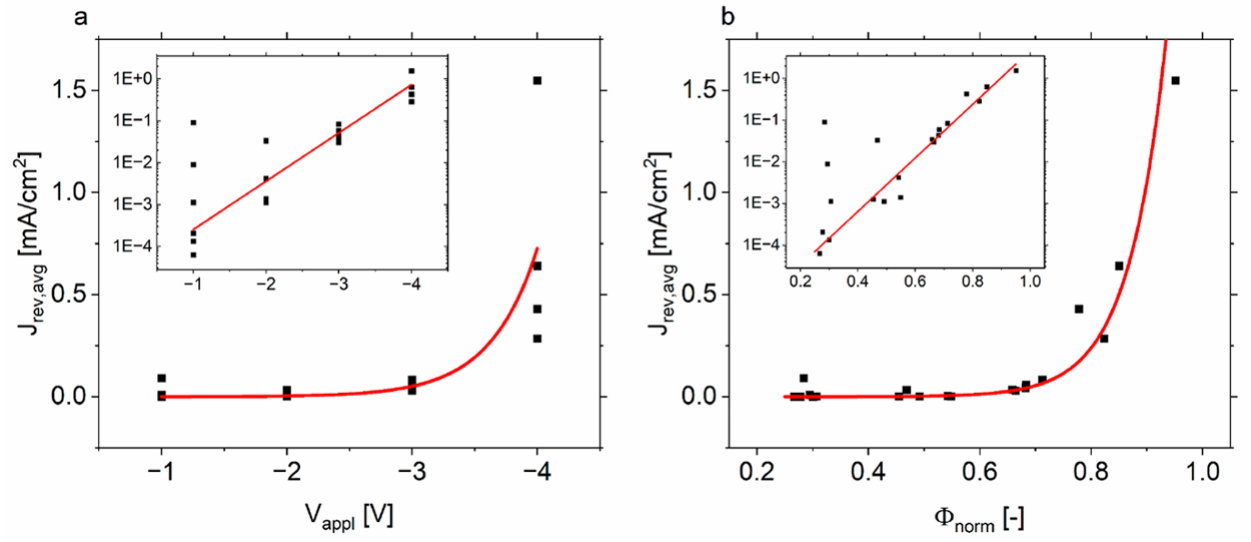
Average reverse bias current densities flowing through perovskite solar cells during the degradation step, plotted against the applied voltage (a) and the normalized voltage (b). The insets show the same on semilogarithmic scales. Exponential functions are inserted as guide to the eye.

By dividing the applied voltage by the breakdown voltage, we obtain the normalized voltage Φ_norm_. If the observed spread is largely caused by the variance of *V*_bd_, we should see a reduced spread when plotting **J**_rev,avg_ against Φ_norm_. This is depicted in Figure 2b where we indeed find a decreased spread at large negative voltages. We see in the insets with semilogarithmic scales that the relationship between *J*_rev,avg_ and Φ_norm_ can now be better approximated by an exponential function.

We only applied reverse biases corresponding to normalized voltages below 1, meaning smaller than the breakdown voltages. This explains the relatively low average current densities, which remained below 1.6 mA/cm^2^. In a simple partial shading event of a perovskite module, we expect a current density that is more than ten times higher (the current that the illuminated cells generate in the maximum-power-point). In the absence of shunts, lower current densities prevent the effects of Joule heating—like absorber decomposition—and shunt formation from hiding other degradation mechanisms, however.

Despite these low average current densities, we observe already significant loss of PCE. Using PCEs from illuminated IV measurements performed directly before and after degradation, we calculate a degradation rate DR by dividing the relative PCE loss by the duration of the degradation *t*_deg_ (see Supporting Information on page S3). Thus, we obtain the degradation rate DR (linearized over time), which is depicted in Figure 3.

**Figure 3:**
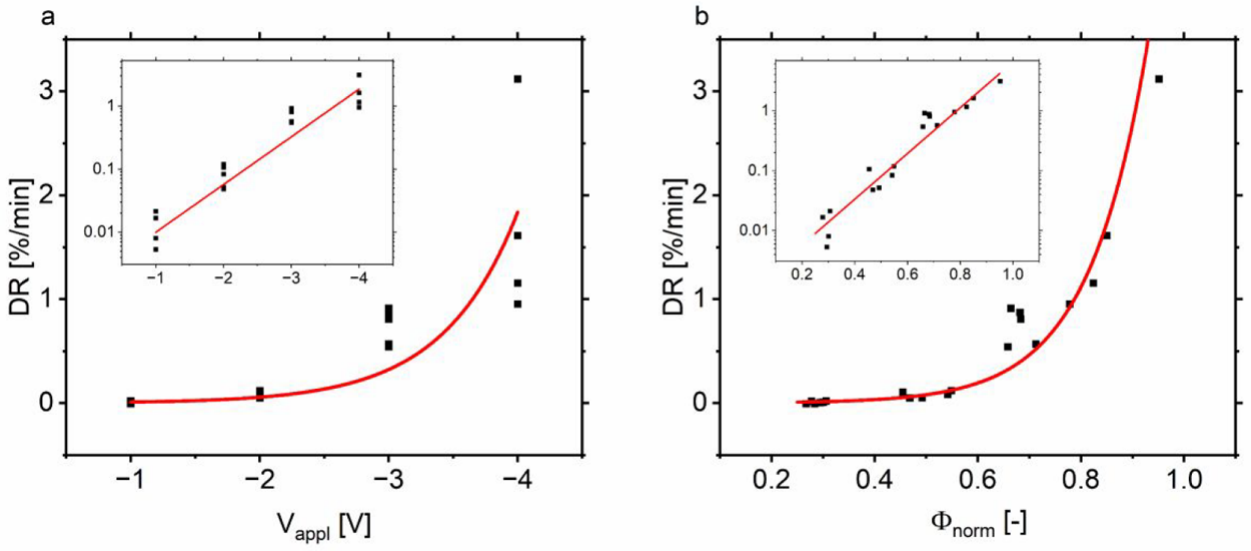
Degradation rate DR plotted against the applied voltage (a) and the normalized voltage (b). The insets show the same on semilogarithmic scales. Exponential functions are inserted as guide to the eye.

Figure 3a shows DR plotted against *V*_appl_ on a linear scale and in the inset on a semilogarithmic scale. We observe a spread that is especially apparent at large reverse biases, as was observed in *J*_rev,avg_. A spread at small voltages like in *J*_rev,avg_, however, is not visible here. Small shunts do not seem to greatly impact the degradation rate, despite their impact on *J*_rev,avg_. If we apply our method of normalizing the applied voltage, we obtain Figure 3b. This brings the data points in an order where higher Φ_norm_ corresponds to higher DR. Most data points can now be well approximated by an exponential function.

Our normalization method revealed that both *J*_rev,avg_ and DR follow a similar dependence on the normalized voltage and that both can be approximated by exponential functions. That both metrics follow a similar dependence is also visible when plotting DR directly against *J*_rev,avg_ (see the Supporting Information on page S5, Figure S2). A linear relationship between the two metrics seems to be present. The conclusion that the dominant degradation mechanisms are current-driven seems natural. In that case, Faraday’s law would govern the relationship between the rates of electrochemical reactions and current, and the reaction rates would be connected to the degradation rates.^15,16^

On the other hand, a voltage-driven process could be accompanied by a current showing a voltage dependence similar to our *J*_rev,avg_. Garcia-Batlle et al. found on monocrystalline perovskites that the ionic current caused by positive voltages can show a superlinear dependence on the voltage.^17^ They, however, also noted that the mobility of holes and electrons is many (∼7–9) orders of magnitude larger than that of ions.^17^ Therefore, the magnitude of the electronic current far outshines the ionic current. The same had already been concluded by Bowring et al. for the reverse bias current.^5^

The degradation rates observed reach up to 3%/min despite the rather low current densities. Using the exponential functions inserted in Figure 2b and Figure 3b and extrapolating to −20 mA/cm^2^, we obtain a DR of about 16%/min at Φ_norm_ ≈ 1.1. After just 3 min, we would have only about half of the initial PCE left. That illustrates once more the importance of understanding the mechanisms behind reverse bias degradation.

Our highest observed DR of 3%/min is also comparable to what has been reported by Jiang et al., who applied a reverse bias of −4 V and saw a decrease of 5% in the first minute.^18^ However, comparing degradation rates at the same applied voltage is difficult if the reverse bias current plays a dominant role. Only knowing the breakdown voltage or information about the current flowing through the device during degradation would allow conclusions about the stability in the case of a partial shading event. This touches on the general problem that there are no standards yet agreed upon for testing and reporting reverse bias degradation of perovskite solar cells beyond the binary answer of IEC 61215. Defining these would allow a comparison of the many different variations of the perovskite solar cell layer stack. This in turn might facilitate the same kind of widespread cooperation that has led to the enormous efficiency increase observed for the technology in the past decade.

We conclude that our results suggest that current-driven degradation mechanisms dominate degradation already at smaller reverse biases. Our method of normalizing the applied voltage with the breakdown voltage revealed similar dependencies of current and degradation rate, which allowed this inference. It highlights the importance of understanding the breakdown and the reverse bias behavior of perovskite solar cells. We additionally propose adding the reverse bias current or the breakdown voltage to reports about the reverse bias degradation of perovskite solar cells to increase comparability.

## Data Availability

The data underlying this study are openly available in 4TU.ResearchData at 10.4121/ef3d2f15-ebf8-48c3-aeb4-629e176f9d05.

## Supporting Information Available

Detailed information about the fabrication of samples and the execution of measurements (PDF)

## References

[ref1] Lan D.; Green M. A. Combatting Temperature and Reverse-Bias Challenges Facing Perovskite Solar Cells. Joule 2022, 6 (8), 1782–1797. 10.1016/j.joule.2022.06.014.

[ref2] Qian J.; Ernst M.; Walter D.; Mahmud M. A.; Hacke P.; Weber K.; Al-Jassim M.; Blakers A. Destructive Reverse Bias Pinning in Perovskite/Silicon Tandem Solar Modules Caused by Perovskite Hysteresis under Dynamic Shading. Sustain. Energy Fuels 2020, 4 (8), 4067–4075. 10.1039/C9SE01246J.

[ref3] Duan L.; Walter D.; Chang N.; Bullock J.; Kang D.; Phang S. P.; Weber K.; White T.; Macdonald D.; Catchpole K.; Shen H. Stability Challenges for the Commercialization of Perovskite-Silicon Tandem Solar Cells. Nat. Rev. Mater. 2023, 8, 261 10.1038/s41578-022-00521-1.

[ref4] Wolf E. J.; Gould I. E.; Bliss L. B.; Berry J. J.; McGehee M. D. Designing Modules to Prevent Reverse Bias Degradation in Perovskite Solar Cells When Partial Shading Occurs. Sol. RRL 2022, 6, 2100239 10.1002/solr.202100239.

[ref5] Bowring A. R.; Bertoluzzi L.; O’Regan B. C.; McGehee M. D. Reverse Bias Behavior of Halide Perovskite Solar Cells. Adv. Energy Mater. 2018, 8 (8), 1702365 10.1002/aenm.201702365.

[ref6] Razera R. A. Z.; Jacobs D. A.; Fu F.; Fiala P.; Dussouillez M.; Sahli F.; Yang T. C. J.; Ding L.; Walter A.; Feil A. F.; Boudinov H. I.; Nicolay S.; Ballif C.; Jeangros Q. Instability of P-i-n Perovskite Solar Cells under Reverse Bias. J. Mater. Chem. A 2020, 8 (1), 242–250. 10.1039/C9TA12032G.

[ref7] Wang C.; Huang L.; Zhou Y.; Guo Y.; Liang K.; Wang T.; Liu X.; Zhang J.; Hu Z.; Zhu Y. Perovskite Solar Cells in the Shadow: Understanding the Mechanism of Reverse-Bias Behavior toward Suppressed Reverse-Bias Breakdown and Reverse-Bias Induced Degradation. Adv. Energy Mater. 2023, 13 (9), 2203596 10.1002/aenm.202203596.

[ref8] Bogachuk D.; Saddedine K.; Martineau D.; Narbey S.; Verma A.; Gebhardt P.; Herterich J. P.; Glissmann N.; Zouhair S.; Markert J.; Gould I. E.; McGehee M. D.; Würfel U.; Hinsch A.; Wagner L. Perovskite Photovoltaic Devices with Carbon-Based Electrodes Withstanding Reverse-Bias Voltages up to −9 V and Surpassing IEC 61215:2016 International Standard. Sol. RRL 2022, 6, 2100527 10.1002/solr.202100527.

[ref9] Bertoluzzi L.; Patel J. B.; Bush K. A.; Boyd C. C.; Kerner R. A.; O’Regan B. C.; McGehee M. D. Incorporating Electrochemical Halide Oxidation into Drift-Diffusion Models to Explain Performance Losses in Perovskite Solar Cells under Prolonged Reverse Bias. Adv. Energy Mater. 2021, 11 (10), 2002614 10.1002/aenm.202002614.

[ref10] Ni Z.; Jiao H.; Fei C.; Gu H.; Xu S.; Yu Z.; Yang G.; Deng Y.; Jiang Q.; Liu Y.; Yan Y.; Huang J. Evolution of Defects during the Degradation of Metal Halide Perovskite Solar Cells under Reverse Bias and Illumination. Nat. Energy 2022, 7 (1), 65–73. 10.1038/s41560-021-00949-9.

[ref11] Gould I. E.; Xiao C.; Patel J. B.; McGehee M. D. In-Operando Characterization of P-I-N Perovskite Solar Cells under Reverse Bias. In Conference Record of the IEEE Photovoltaic Specialists Conference; IEEE, 2021; pp 1365–1367. 10.1109/PVSC43889.2021.9518723.

[ref12] Li W.; Huang K.; Chang J.; Hu C.; Long C.; Zhang H.; Maldague X.; Liu B.; Meng J.; Duan Y.; Yang J. Sparkling Hot Spots in Perovskite Solar Cells under Reverse Bias. ChemPhysMater. 2022, 1 (1), 71–76. 10.1016/j.chphma.2021.10.001.

[ref13] Bracesco A. E. A.; Burgess C. H.; Todinova A.; Zardetto V.; Koushik D.; Kessels W. M. M.; Dogan I.; Weijtens C. H. L.; Veenstra S.; Andriessen R.; Creatore M. The Chemistry and Energetics of the Interface between Metal Halide Perovskite and Atomic Layer Deposited Metal Oxides. J. Vac. Sci. Technol. A 2020, 38 (6), 63206 10.1116/6.0000447.

[ref14] Głowienka D.; Zhang D.; Di Giacomo F.; Najafi M.; Veenstra S.; Szmytkowski J.; Galagan Y. Role of Surface Recombination in Perovskite Solar Cells at the Interface of HTL/CH_3_NH_3_PbI_3_. Nano Energy 2020, 67, 104186 10.1016/j.nanoen.2019.104186.

[ref15] Faraday M. VI. Experimental Researches in Electricity.-Seventh Series. Philos. Trans. R. Soc. London 1834, 124, 77–122. 10.1098/rstl.1834.0008.

[ref16] Jensen W. B. Faraday’s Laws or Faraday’s Law?. J. Chem. Educ. 2012, 89 (9), 1208–1209. 10.1021/ed101193q.

[ref17] García-Batlle M.; Mayén Guillén J.; Chapran M.; Baussens O.; Zaccaro J.; Verilhac J.-M.; Gros-Daillon E.; Guerrero A.; Almora O.; Garcia-Belmonte G. Coupling between Ion Drift and Kinetics of Electronic Current Transients in MAPbBr 3 Single Crystals. ACS Energy Lett. 2022, 7 (3), 946–951. 10.1021/acsenergylett.1c02578. 35310458 PMC8922277

[ref18] Jiang C.; Zhou J.; Li H.; Tan L.; Li M.; Tress W.; Ding L.; Grätzel M.; Yi C. Double Layer Composite Electrode Strategy for Efficient Perovskite Solar Cells with Excellent Reverse-Bias Stability. Nano-Micro Lett. 2023, 15 (1), 1–11. 10.1007/s40820-022-00985-4. PMC9747998 36512180

