# Supplementary material for: Impact of the Current on Reverse Bias Degradation of Perovskite Solar Cells
Source: ACS Appl Energy Mater. 2023 Nov 10;6(22):11429–32. doi: 10.1021/acsaem.3c02273 (PMC10685325; doi:10.1021/acsaem.3c02273)
Supplement: Supplementary file 1 — ae3c02273_si_001.pdf [file ae3c02273_si_001.pdf]

Supporting Information

The impact of the current on reverse bias degradation of perovskite solar cells

Jonathan Henzel[1,2], Klaas Bakker[1], Mehrdad Najafi[1], Valerio Zardetto[1], Sjoerd Veenstra[1], Olindo Isabella[2], Luana Mazzarella[2], Arthur Weeber[1,2], and Mirjam Theelen[1]*

1: TNO, partner in Solliance, High Tech Campus 21, 5656 AE Eindhoven, The Netherlands.

2: Delft University of Technology, Photovoltaic Materials and Devices, Mekelweg 5, 2628 CD Delft, The Netherlands.

*Corresponding author. E-mail: jonathan.henzel@tno.nl

Details about fabrication and layer stack

The layer stack on the glass superstrate is listed in Table S1 with the approximate layer thicknesses. The cell area is 0.16 cm^2. The order of deposition is from the top to the bottom of the table. ITO stands for Indium-Tin Oxide and PTAA for Poly(triaryl amine).

Table S1: The layer stack listed with thicknesses and deposition method on top of the glass substrate.

**Layer material**	**Thickness [nm]**	**Deposition process**
ITO	130	Sputtering
PTAA	10	Spin coating
Perovskite (E_g = 1.6 eV)	600	Spin coating with gas quenching
C_{60}	20	Thermal evaporation
SnO_2	45	Spatial ALD
ITO	180	Sputtering

Glass superstrates (3 cm x 3 cm) with patterned ITO are cleaned with soap water, de-ionized water and isopropanol, respectively by hand and by sonication. After the last cleaning step, they are treated in a UV/ozone oven for 30 min. After transport into an N_2-filled glovebox, a PTAA solution (SigmaAldrich, 2 mg/ml in Toluene) is deposited via spin-coating (5000 rpm, 3000 rpm/s, 35 s). The samples are annealed for 10 min on a 100 °C hotplate. A 1.4M perovskite solution ($Cs_{0.05}MA_{0.15}FA_{0.8}PbI_{2.7}Br_{0.3}$), prepared from PbI_2 (Alfa Aesar), $PbBr_2$ (SigmaAldrich), $MABr_2$ (greatcell), FAI (TCI) and CsI (SigmaAldrich) in DMF/NMP (9:1), is spin-coated dynamically (first 2000 rpm, 200 rpm/s for 10 s, then 5000 rpm, 2000 rpm/s for 30 s) with gas-quenching after 15 s. MA stands for methylammonium and FA for formamidinium. The samples are then placed on a hotplate (130 °C) for 10 min. A C_{60}-layer is added via evaporation, a SnO_2-layer via spatial ALD and the patterned ITO electrode on top via sputtering. Gold fingers at the edge of the cell areas are deposited via evaporation. The bottom ITO electrode is revealed by removing all layers with a cotton bud and a DMF:chlorobenzene solution (1:6). Lastly, silver paste is applied to the contacting areas at the edges of the glass superstrates. Four cells are fabricated on one superstrate.

The cells were not packaged.

Details about the experimental procedure

Every cell passed through the same experimental procedure that consisted of a first characterization step, the degradation step, followed by a second characterization step. Each characterization step included (illuminated) IV measurements, dark IV measurements, dark IV measurements with extended voltage range, and maximum-power-point-tracking. During the degradation step, a constant reverse bias was applied in the dark and the current measured. All measurements were performed in air and at room temperature. During light IV measurements and MPPT, dry air was blown over the cells for cooling. Detailed information is collected in Table S2.

Table S2: Details about the experimental procedure.

**Measurement**	**Measurement tool**	**Additional information:**
IV measurement	Dual light source simulator (WACOM, AM1.5G), Keithley 2400 source meter, illumination mask 0.09 cm^2	forward/reverse sweep: [1.2 V;-0.5 V], step: 0.02 V, scan rate: 0.1 V/s
Dark IV measurement (DIV)	Keithley 2400 source meter	forward/reverse sweep: [1.2 V;-0.5 V], step: 0.02 V, scan rate: 0.1 V/s
Extended dark IV measurement (DIV_{ext})	Autolab PGSTAT30, NOVA 2.1.4	forward/reverse sweep: [1 V;-8 V] with current cut-off at first at 1.75 mA then at 1 mA, step: 0.01 V, scan rate: 0.2 V/s
Maximum-power-point-tracking (MPPT)	Dual light source simulator (WACOM, AM1.5G), Keithley 2400 source meter, illumination mask 0.09 cm^2	Measurement frequency: 0.32 Hz
Degradation step/ reverse bias current measurement	Autolab PGSTAT30, NOVA 2.1.4	Measurement frequency: 4 Hz

Definitions of metrics

We calculate the average reverse bias current density from the reverse bias current $I_{rev}(t)$ that we measure during a degradation step with the duration t_{deg}. A_{cell} is the cell area. Then, the average reverse bias current flowing through the cell is:

$$J_{rev,avg} = \frac{\int_0^{t_{deg}} I_{rev}(t)dt}{A_{cell} \cdot t_{deg}}.$$

We calculate a degradation rate from the PCEs before (PCE_{in}) and after (PCE_{fin}) degradation and the duration of the degradation step t_{deg}:

$$DR = \left(\frac{PCE_{in} - PCE_{fin}}{PCE_{in}}\right) / t_{deg}.$$

The spread of the breakdown voltage

In Figure S1, the results of dark IV measurements with extended voltage range (DIV_{ext}) are shown. At the current density of -1 mA/cm^2, a horizontal line marks the breakdown voltage (V_{bd}). The current density shows hysteretic behavior in the reverse bias regime. For each cell, the mean of the breakdown voltages of reverse and forward sweep was used. V_{bd} varies among the cells of this batch between - 3.3 V and - 5.1 V.

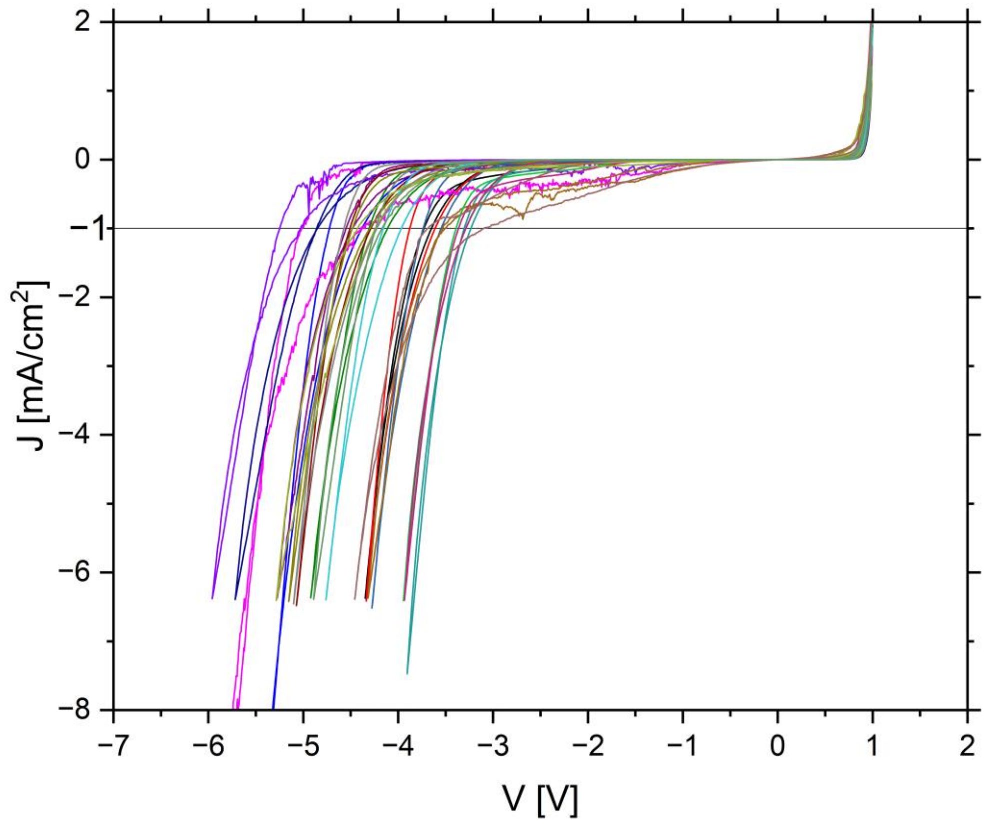

Figure S1: The current density curves of 20 cells from dark IV measurements with extended voltage range (DIV_{ext}). The breakdown voltage is at the intersection with the horizontal line at - 1 mA/cm^2.

The relation of the degradation rate with the average current density

The observation that both DR and $J_{rev,avg}$ show a similar (exponential) dependence on the normalized voltage can be visualized by plotting DR directly against $J_{rev,avg}$. A similar dependence would then lead to a linear dependence of the two metrics on each other. This is visible in Figure S2. However, due to the nature of the experiment, the data points are largely clustered at small values for $J_{rev,avg}$ where a significant spread is apparent.

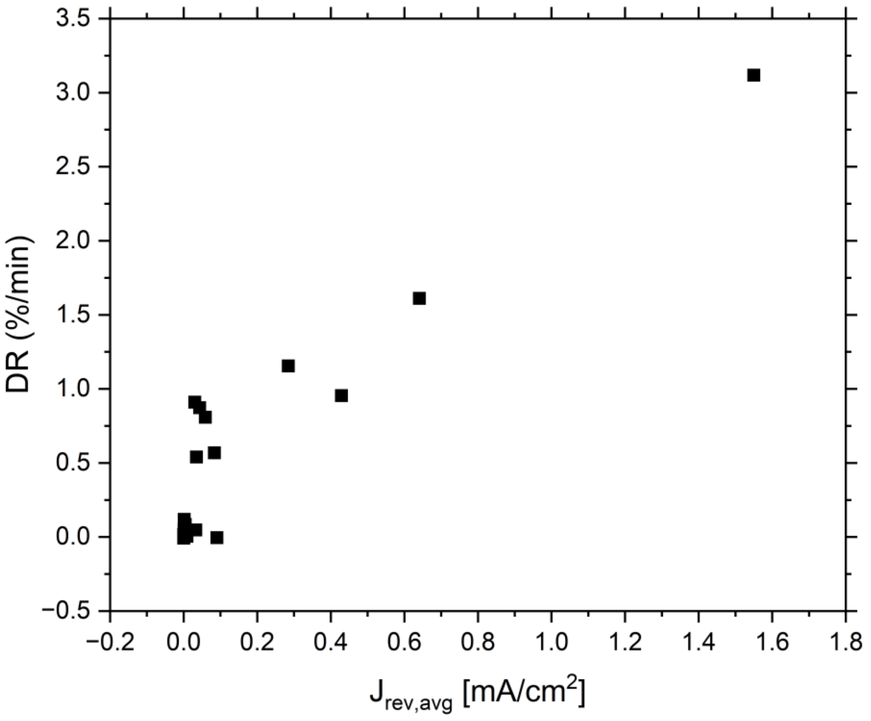

Figure S2: The degradation rate DR is plotted against the average current density $J_{rev,avg}$.
